# Supplementary material for: A Global Screen for Assembly State Changes of the Mitotic Proteome by SEC-SWATH-MS
Source: Cell Syst. 2020 Feb 26;10(2):133–155.e6. doi: 10.1016/j.cels.2020.01.001 (PMC7042714; doi:10.1016/j.cels.2020.01.001)

Q15056 | IF4H_HUMAN | EIF4H KIAA0038 WBSCR1 WSCR1
Monomer MW [kDa]: 27.385 Monomer expected elution fraction: 51

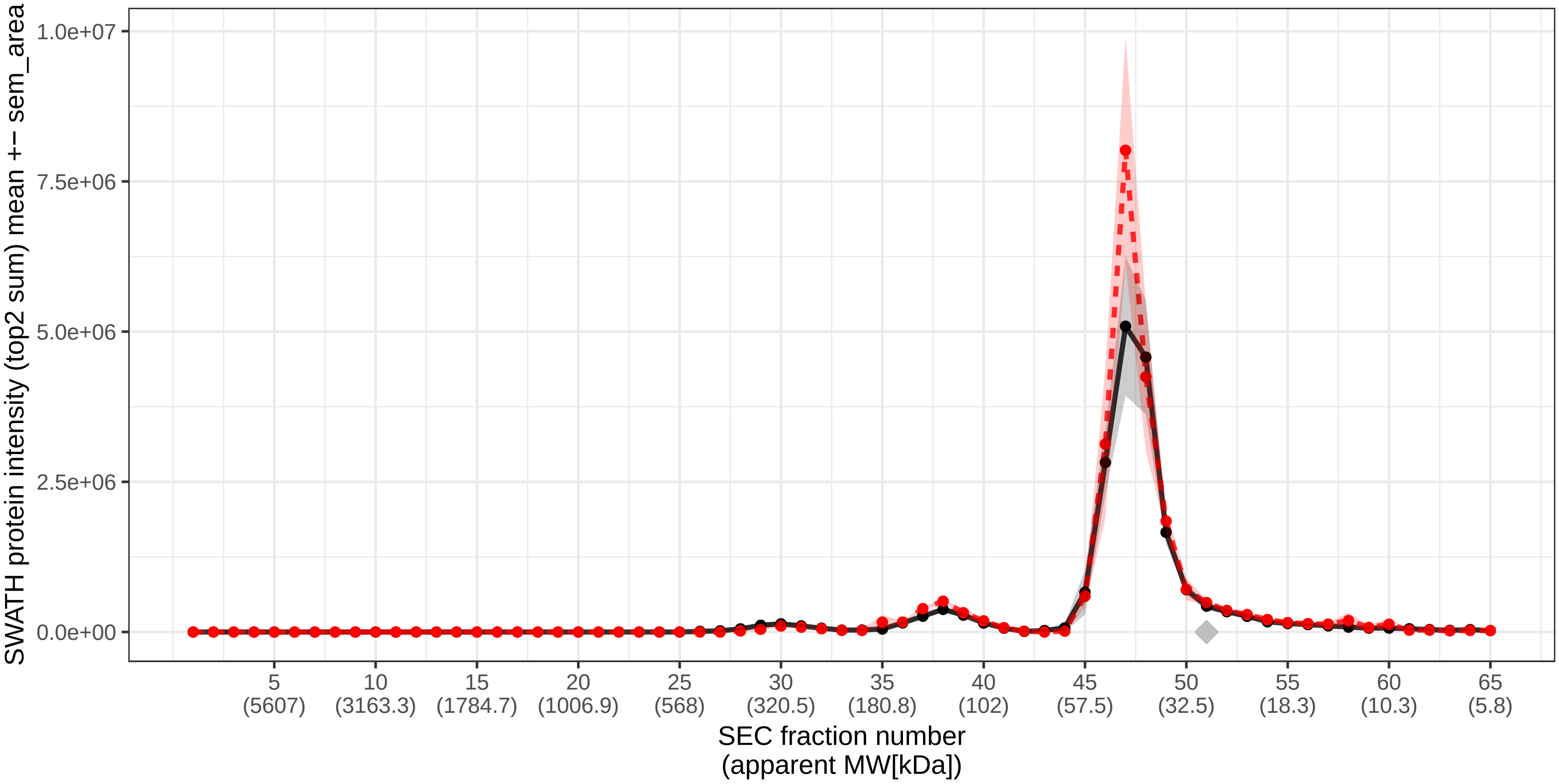

Supplement: Data S1. SEC-SWATH-MS Protein Chromatograms, Related to Figure 1 [file mmc6.zip › SECchrom_Q15056_IF4H_HUMAN_EIF4H_KIAA0038_WBSCR1_WSCR1.pdf]
